# The strategic case for establishing public-private partnerships in cancer care

**DOI:** 10.1186/s12961-015-0031-x

**Published:** 2015-10-14

**Authors:** Debra J. Holden, Kristin Reiter, Donna O’Brien, Kathleen Dalton

**Affiliations:** RTI International, 3040 E Cornwallis Rd, PO Box 12194, Research Triangle Park, NC 27709 USA; Department of Health Policy and Management, The University of North Carolina, Chapel Hill, NC 27599 USA; Strategic Visions in Healthcare, LLC, New York, NY USA

**Keywords:** Cancer care, Partnerships, Program evaluation, Strategic case

## Abstract

**Background:**

In 2007, the National Cancer Institute (NCI) launched the NCI Community Cancer Centers Program (NCCCP) as a public-private partnership with community hospitals with a goal of advancing cancer care and research. In order to leverage federal dollars in a time of limited resources, matching funds from each participating hospital were required. The purpose of this paper is to examine hospitals’ level of and rationale for co-investment in this partnership, and whether there is an association between hospitals’ co-investment and achievement of strategic goals.

**Methods:**

Analysis using a comparative case study and micro-cost data was conducted as part of a comprehensive evaluation of the NCCCP pilot to determine the level of co-investment made in support of NCI’s goals. In-person or telephone interviews with key informants were conducted at 10 participating hospital and system sites during the first and final years of implementation. Micro-cost data were collected annually from each site from 2007 to 2010. Self-reported data from each awardee are presented on patient volume and physician counts, while secondary data are used to examine the local Medicare market share.

**Results:**

The rationale expressed by interviewees for participation in a public-private partnership with NCI included expectations of increased market share, higher patient volumes, and enhanced opportunities for cancer physician recruitment as a result of affiliation with the NCI. On average, hospitals invested resources into the NCCCP at a level exceeding $3 for every $1 of federal funds. Six sites experienced a statistically significant change in their Medicare market share. Cancer patient volume increased by as much as one-third from Year 1 to Year 3 for eight of the sites. Nine sites reported an increase in key cancer physician recruitment.

**Conclusions:**

Demonstrated investments in cancer care and research were associated with increases in cancer patient volume and perhaps in recruitment of key cancer physicians, but not in increased Medicare market share. Although the results reflect a small sample of hospitals, findings suggest that hospital executives believe there to be a strategic case for a public-private partnership as demonstrated through the NCCCP, which leveraged federal funds to support mutual goals for advancing cancer care and research.

## Background

Cancer is a complex disease that requires broad engagement of various clinical specialists and organizations to develop a patient-centered delivery system that integrates scientific discovery and provides highly coordinated, evidence-based multi-specialty care across the care continuum. While these systems are well-established in many of the largest cancer centres in the United States, not all patients have access to these centres. Rather, the majority of cancer patients in the United States receive care in their local communities. To promote access to the same high-quality care for patients, regardless of residence, the National Cancer Institute (NCI), one of the 27 institutes of the National Institutes of Health, designed the Community Cancer Centers Program (NCCCP) pilot as a public-private partnership to accelerate the diffusion of the latest science to local communities.

In a public-private partnership, government agencies and private sector organizations share in the financing and operations of a program or project to accomplish important public goals [1,2]. In the case of the NCCCP, the federally-funded NCI partnered with not-for-profit community hospitals to share risk and cost, as well as knowledge and capabilities to advance cancer care in six key areas: disparities, clinical trials, biospecimens, information technology, quality of care, and survivorship and palliative care [3-5]. Through the NCCCP, the NCI provided funding to eight community hospitals and two multi-hospital systems, each operating a cancer program and caring for at least 1,000 new cancer patients annually. Each site was awarded approximately US$ 500,000 per year to cover costs for specific deliverables related to the NCCCP award. In addition to receiving federal funds, each organization was expected to contribute institutional dollars (i.e. matching funds) to cover NCCCP-related activities including redesigning care processes and organizational structures; however, the amount of matching funds was not specified.

The success of public-private partnerships such as the NCCCP rests with the commitment of executive leaders at private sector (non-government) organizations to invest time and resources into the required activities. However, challenges exist when resources are constrained, and financial benefits cannot be demonstrated. Leatherman et al. [6] demonstrated the effects of financing misalignments on quality initiatives, defining the concepts of the business, economic, and social cases for quality of care. They argued that private sector organizations were unlikely to sustain quality improvement activities in the absence of a demonstrated business case: either a short-term, positive financial return on an activity or the expectation of positive changes to organizational function and sustainability.

Previous studies examining quality improvement initiatives in cancer have focused on the business case [7,8], and researchers have struggled to find hard evidence of a quantifiable financial return on investment. However, leaders continue to invest in clinical programs such as cancer care, citing indirect benefits such as reputational effects and the ability to provide access to cutting-edge care. These sorts of qualitative considerations have been shown to play a significant role in decision making around investments by not-for-profit hospitals [8-11]. As a result, the concept of a business case may not fully capture all of the perceived benefits of an investment to improve a cancer program. Therefore, for this study, we defined the “strategic case” for NCCCP participation.

In our definition, the strategic case encompasses the business, economic, and social cases and includes those outcomes of greatest interest to the hospital executive management. Strategic outcomes are those that drive management decisions in how best to allocate program-specific resources. This paper presents study findings that explore (1) the extent to which hospital executive management believed there to be a strategic case for participation and the specific outcomes that comprised the strategic case, (2) the level of investments made by the participating hospitals, and (3) whether the total investments were associated with changes in the strategic outcomes specified by hospital executive management.

## Methods

NCI contracted with RTI International (RTI) to conduct a comprehensive evaluation of the NCCCP pilot [12], including a micro-cost study and a comparative case study [5]. Although a total of 16 hospitals implemented the NCCCP, only the 10 lead sites are the focus of the evaluation findings reported herein. In addition to these components of the independent evaluation, research projects were conducted by leaders from the program [13-17], or were added as evaluation methods [18,19].

### Data collection and secondary sources

The evaluation collected data on NCCCP-related spending as well as qualitative data from interviews with hospital executive management to gain insights into how executives view the financial and other (strategic) benefits associated with NCCCP participation and how they weigh those benefits against the costs. RTI conducted initial and follow-up interviews with Chief Financial Officers (CFOs) by phone and with Chief Executive Officers (CEOs) or Chief Operating Officers (COOs) during site visits each year to participating hospitals. Selected anonymous quotes from these interviews are presented in italics and identified as being stated by a CFO or a CEO/COO during the specified year of program implementation.^a^

Micro-cost data were collected each year on an Excel-based input document called the Cost Assessment Tool [20-22]. The tool was customized to capture spending across three cost domains for each year of program implementation: NCI-funded subcontract costs (invoiced costs), costs underwritten by the sponsoring organization (matching costs), and a valuation of donated time (donated costs).^b^ In addition to these primary data sources, numerous secondary data sources were used to examine elements of the context within which each site was operating. In this paper, we present findings from the following secondary sources.

#### Assessment surveys

Annual surveys completed by each participating hospital that collected variables such as unduplicated cancer patient volume ^c^ and the number of key cancer physicians that operate in the cancer centre or program.

#### Medicare market share

The market of the NCCCP hospitals was defined as the Core Based Statistical Area in which the hospital is located and identification of other hospitals located within the NCCCP markets obtained from the Annual Survey of the American Hospital Association data. For all hospitals in each NCCCP market, the total number of Medicare discharges in the hospital during Fiscal Years (FYs) 2006 and 2011 was determined using the “bills” variable from the annual Medicare Inpatient Prospective Payment System.^d^ For each FY and each hospital market, total Medicare discharges from market hospitals were summed to determine the total number of Medicare discharges. The NCCCP hospital’s share of Medicare discharges was calculated as the proportion of the market’s discharges that were from that hospital.

### Analysis strategy

Qualitative data were coded and analysed using state-of-the-art procedures to identify thematic findings characterising the strategic outcomes valued by hospital executives [23]. To assess levels of hospital co-investment, micro-cost data were analysed using Stata software and the methods described in the Economic Evaluation Report [20]. To assess whether there were changes over time in any of the three strategic outcomes – patient volume, market share, and physician recruitment – identified through the interviews, we collected secondary data from the sources described above and tested for statistically significant differences between FY 2006 and FY 2011 using *t*-tests for samples of equal variance. Finally, to assess whether levels of investment were associated with performance on any of the strategic outcomes, data for the three outcomes were ranked according to the percent change for a hospital site, specific to each variable. This allowed consideration of the variation in starting points for the hospitals. The percent change from FY 2006 to FY 2011 was rank ordered such that those hospitals that were among the highest achievers (compared to the other nine NCCCP sites) for that outcome received a rank of 1, and the lowest received a rank of 10. These ranks were then categorized as either “high” for the top five sites or “low” for the bottom five sites to explore patterns across sites on how they performed on each outcome relative to their total investments in the NCCCP.

In this paper, we first present perceptions of the strategic case as described by CFOs and CEOs or COOs, and descriptive analysis of changes over time in the three strategic outcomes. We then present overall cost study data for each site across the 3 years of the pilot program. Finally, we examine the extent to which the three key strategic outcomes were achieved by the sites (i.e. increases in Medicare market share, cancer patient volume, and key cancer physicians) relative to hospital investment levels.

## Results

### Relevant hospital characteristics

During the process of site selection for the NCCCP pilot, NCI purposefully selected hospitals that had varying levels of development of their cancer programs or service lines [3]. Several sites had limited capacity or history with conducting clinical trials research, working with disparate populations, and/or coordinating care across the cancer continuum to ensure that patients received evidence-based, timely care. Across the participating hospitals, the position of the cancer program within the organizational structure also differed (Holden, et al., 2012). For example, some hospitals had independent cancer service lines with a physician director reporting directly to hospital executive, whereas others were much more fragmented organisationally. Eight of the lead sites reported having a physician director for the cancer centre or program as of 2007, with varying degrees of time devoted to this position. All but two of the 10 lead sites relied primarily on cancer physicians working in private practices. All lead sites had to have at least 1,000 new cancer patients in CY 2006 in order to qualify for NCCCP [12].

### Overview of top hospital management interview findings and changes in outcomes

CEOs/COOs and CFOs tended to stress different reasons for NCCCP participation. For example, CEOs/COOs tended to emphasize how the program would help them improve quality of cancer care and enhance their image in cancer research and care, whereas CFOs tended to emphasize how the program would help them to become a leader in their local market. However, all consistently agreed that there were four primary motivations that provide a strategic case for their involvement: (1) increases in their market share for cancer care, particularly through the affiliation with NCI; (2) increases in their patient volume and enhancement of their payer mix; (3) recruitment of key cancer physicians; and (4) increases in access to care and/or research for local cancer patients. Although many sites found it difficult to separate the effects of the NCCCP from other efforts related to cancer care, virtually all CFOs recognized the strategic fit of the NCCCP with quality improvement goals, and the reputational value of formal affiliation with NCI. For example, two separate CFOs commented:

“*Volume growth, contribution to margin increase, and recruitment of physicians and retention are things that we are tracking for our strategic program and for an entire service line.*” (CFO, Year 1)

“*We also saw the NCI connection as a way to differentiate ourselves from other community hospitals out there who are competitors…*” (CFO, Year 1)

### Market share

As shown in Table 1, sites varied in the extent to which they led their local market for Medicare patients as of 2006, ranging from 1.72% (Site 2), indicating a highly competitive market, to 80.57% (Site 8), a market leader. CFOs across the sites consistently viewed the NCCCP as providing them with a mechanism for becoming a market leader for cancer care:

**Table 1:** **Results of Medicare market share at baseline and follow-up of NCCCP implementation**

**Site number** ^**a**^	**Medicare market share (2006)** ^**b**^ **%**	**Medicare market share (2011)** ^**c**^	**Differences**	**Ranking of differences** ^**d**^	**Degree of change** ^**e**^
Site 1	30.97	31.21	0.24	3	H
Site 2	1.72	1.54*	−0.18	5	H
Site 3	46.54	45.60	−0.94	9	L
Site 4	50.61	54.60*	3.99	1	H
Site 5	15.45	14.69*	−0.76	7	L
Site 6	8.85	8.93	0.08	4	H
Site 7	7.51	5.05*	−2.46	10	L
Site 8	80.57	80.01	−0.56	6	L
Site 9	48.22	47.43	−0.79	8	L
Site 10	25.20	26.42*	1.22	2	H

*P <0.01.

^a^Sites have been randomly numbered to ensure confidentiality of data.

^b^Data Source: Medicare Provider of Service file.

^c^Data Source: Medicare Provider of Service file.

^d^1 = highest positive change; 10 = lowest positive change or highest negative change.

^e^H = high degree of change; L = low degree of change.

“*We are one of the leaders in the market from a cardiology standpoint, surgical standpoint, and orthopedic standpoint, but in oncology we are not. That is one of the reasons why we are interested in this particular program…We view these as important value-added activities and view the payoff* [as] *coming from the additional business that is provided…and attracting the payer mix that will provide the funding to do these kinds of things.*” (CFO, Year 1)

“*I think reputation and market share: those are measures that show we are doing something right in the community…*” (CFO, Year 1)

As shown in Table 1, Medicare market share in 2011 for the 10 lead sites had changed to some extent, ranging from 1.54% (Site 2) to 80.01% (Site 8). Over the 5-year period, six sites experienced a statistically significant change in their Medicare market share, although the increases cannot be causally linked to the NCCCP.

### Cancer patient volume

Becoming a market leader may not, in and of itself, be sufficient to maintain the NCCCP beyond the funding period, especially in the face of fiscal challenges. Respondents noted that the strategic case for NCCCP participation also involved increasing patient volume, along with enhancing their payer mix:

“[The NCCCP] *has been a catalyst for us to raise the bar. It drives growth. We want to have more patients go through our program to show those outcomes, because greater numbers prove a lot more…*” (CEO/COO, Year 1)

“*Patients choosing to come here – because we offer complete and state-of-the-art cancer care services – is critical… We benefit financially from that, because it is one of our more profitable service lines.*” (CFO, Year 1)

Although enhanced reputation is difficult to assess, the overall evaluation did capture the extent to which hospitals changed their cancer patient volume. Although a causal link to NCCCP participation cannot be established, data reported by sites in their initial proposals for funding compared to their Final Assessment Survey (specific to NCCCP) suggest the unduplicated cancer patient volume increased by about one-third from Year 1 to Year 3 for four sites (percentage increases of 29.1% to 36.8%); the remaining four sites reported increases ranging from 1.9% to 20.1% (Table 2). Sites 3 and 4 experienced a decrease in their cancer patient volume during the course of the pilot (−18.3% and −0.3%, respectively).

**Table 2:** **Results of cancer patient volume at baseline and follow-up of NCCCP implementation**

	**Total cancer patient volume**					
**Site number** ^**a**^	**Baseline (CY 2006)** ^**b**^	**Follow-up (CY 2009)** ^**c**^	**Net change**	**Percent change (%)**	**Ranking of change** ^**d**^	**Degree of change** ^**e**^
Site 1	1,057	1,400	343	32.4	3	H
Site 2	1,527	1,577	50	3.3	7	L
Site 3	2,591	2,116	475	−18.3	10	L
Site 4	1,429	1,424	−5	−0.3	9	L
Site 5	2,290	3,133	843	36.8	1	H
Site 6	2,863	3,133	280	9.8	6	L
Site 7	1,075	1,464	389	36.2	2	H
Site 8	1,379	1,781	402	29.1	4	H
Site 9	1,236	1,484	248	20.1	5	H
Site 10	2,595	2,645	50	1.9	8	L

^a^Sites have been randomly numbered to ensure confidentiality of data.

^b^Data Source: Initial Request for Proposals responses from sites for Calendar Year (CY) 2006.

^c^Data Source: Final Assessment Survey collected for CY 2009.

^d^1 = highest positive change; 10 = lowest positive change or highest negative change.

^e^H = high degree of change; L = low degree of change.

### Key cancer physician recruitment

As the hospitals grew in their local reputations for cancer care through NCCCP participation, the consistent hope among hospital executive management was that this would also enhance their ability to recruit key cancer physicians. This enhanced recruitment would be performed through their association with NCI and the obvious priority they were placing on their cancer service line as well as through their added capacity to conduct cancer research, something that many noted was important to their physicians:

“*The ability to recruit specialists is really important to us, and we haven’t really tried to quantify that, but that is important.*” (CFO, Y1)

“*The connection to NCI is very important.* [It] *helps us recruit new physicians and helps us recruit excellent nurses. People want to be a part of a special program. The linkage is pretty compelling.*” (CEO/COO, Year 3)

While full attribution to the NCCCP is not possible, all but one site (Site 7) reported an increase in the number of key cancer physicians available to their program, with additions for the nine remaining hospitals ranging from as high as 35 to as low as 1 (Table 3). Anecdotally, we know that two hospitals recruited new cancer physician directors during the first year of the program, each of whom told us in later years that they came to their hospital largely because of the NCI connection and the NCCCP.

**Table 3:** **Results of key cancer physician recruitment at baseline and follow-up of NCCCP implementation**

	**Number of key cancer physicians** ^**a**^				
**Site number** ^**b**^	**Baseline (CY 2006)** ^**c**^	**Follow-up (CY 2009)** ^**d**^	**Change**	**Ranking of change**	**Degree of change** ^**e**^
Site 1	8	11	+3	8	L
Site 2	4	39	+35	1	H
Site 3	32	37	+5	5	H
Site 4	10	11	+1	9	L
Site 5	9	18	+9	3	H
Site 6	25	30	+5	5	H
Site 7	12	10	−2	10	L
Site 8	10	16	+6	4	H
Site 9	17	38	+19	2	H
Site 10	19	24	+5	5	H

^a^Sites were asked to provide the “number and type of clinical staff” that “operate in the cancer centre [as of a given date] as noted in the definition provided.” For this variable, sites were to indicate the number and type of staff such as medical oncologists, radiation oncologists, and clinical research nurses. From this information, the number of physicians was extrapolated. The count presented excludes physician assistants, physicists, dosimetrists, therapists, technicians, geneticists, phlebotomists, or any other role clearly not a physician.

^b^Sites have been randomly numbered to ensure confidentiality of data.

^c^Data Source: Baseline Assessment Survey (BAS) collected for Calendar Year (CY) 2006.

^d^Data Source: FAS collected for CY 2009.

^e^1 = highest positive change; 10 = lowest positive change or highest negative change. Note that three hospitals reported a positive change of five additional key cancer physicians so are ranked as ties, skewing the count to seven “high” ranked sites.

### Micro-cost data findings

As revealed through the hospital executive management interviews, site leaders recognized the need to invest in the program and the components that were important to NCI, committing to co-investment at the time of program initiation. Across the 3 years of the pilot, the 10 lead sites reported a total of nearly US$ 37 million of their own investment as matched to less than US$ 15 million received from NCI. In terms of donated costs, sites reported a total of 78,634 hours. Reported donated time varied across sites from less than 400 hours (Site 6) to more than 24,000 hours (Site 8). The following sections provide a summary of variations in the costs by year and site.

### Costs across pilot implementation Years 1–3

The NCCCP was very successful in leveraging government dollars, both by direct site investment and by donated services (Table 4). We define leverage as the value of these co-investments (i.e. private funds) expressed relative to the value of NCCCP funding (i.e. public funds). Per dollar of NCCCP funds distributed to sites, the overall leverage was US$ 36.8 million across all 3 years of pilot implementation. Findings indicate further that for every US$ 1 received by NCI each year, sites invested a total of US$ 3.47 in Year 1, US$ 3.66 in Year 2, and US$ 4.00 in Year 3, including both matching and donated costs.

**Table 4:** **Additional spending per dollar of total NCCCP funding, by site**

**Site**	**Invoiced dollar (US$)**	**Matching dollar (US$)**	**Donated time dollar (US$)**	**Total additional investment (US$)**	**Ranking** ^**a**^	**Degree of change**
Site 1	1.00	2.91	2.33	5.24	2	H
Site 2	1.00	3.05	0.52	3.57	5	H
Site 3	1.00	1.81	1.10	2.91	7	L
Site 4	1.00	0.84	0.04	0.88	10	L
Site 5	1.00	1.95	1.05	3.00	6	L
Site 6	1.00	1.18	0.05	1.23	9	L
Site 7	1.00	4.25	0.46	4.72	4	H
Site 8	1.00	6.06	1.41	7.47	1	H
Site 9	1.00	4.67	0.42	5.09	3	H
Site 10	1.00	2.35	0.12	2.46	8	L
Average, all sites	1.00	3.29	0.85	4.14		

^a^Ranking is across the sites, by total additional investments, from highest (1) to lowest (10).

Source: RTI Analysis of Completed Cost Assessment Tools, Years 1–3.

### Co-investment by site

The biggest component of both matching and donated costs reported by sites every year was physician time. Although the share of total spending by cost domain varied across sites, some sites showed more consistent spending patterns over time than others. As shown in Table 4, sites ranged from investing as little as US$ 0.88 for every US$ 1 received from NCI (both matching and donated costs) to as high as US$ 7.47, with an average additional investment across all 3 years of US$ 4.14.

### Association of co-investment with evidence of reaching desired strategic goals

We explored whether there was an association between levels of co-investment and the extent to which hospitals experienced changes in three strategic outcomes identified during interviews with executives: increases in Medicare market share, cancer patient volume, and/or number of key cancer physicians. As shown in Table 5, cancer patient volume seemed to be more closely associated with total investments than the other two outcomes, with four sites reporting the highest degree of change in cancer patient volume paired with the highest level of investments and four other sites reporting the opposite (low volume and low investments relative to other sites). There did not seem to be an association between positive Medicare market share change and investments. Findings specific to increases in number of key cancer physicians are difficult to determine because all but one site experienced an increase.

**Table 5:** **Examination of the association between site total co-investments in NCCCP and changes in desired outcomes: increased market share, cancer patient volume, and number of key cancer physicians**

**Total investments (matching + donated costs)**	**Medicare market share (change from year 1 to year 3)**		**Cancer patient volume (change from year 1 to year 3)**		**Number of key cancer physicians (change from year 1 to year 3)**	
	**Low**	**High**	**Low**	**High**	**Low**	**High**
Matching + Donated costs: LOW TOTAL	2	4	4	1	1	4
Matching + Donated: HIGH TOTAL	2	2	1	4	2	3

## Discussion

One of the most innovative aspects of the NCCCP is the fact that it was established as a public-private partnership between the federal government and numerous not-for-profit hospitals [1,3]. With significant decreases in funding from both the private and public sectors, the need for such collaborations is paramount to improving health quality while also reducing costs [1,3,24]. To establish this partnership, NCI provided each selected hospital with a relatively small amount of funding (approximately US$ 500,000 per year for each of the 10 funded sites), and any remaining resources needed to meet NCCCP deliverables were required as matching or donated funds from participating hospitals. Micro-cost study findings indicate that, by the end of Year 3, on average, for every US$ 1 provided by NCI, participating hospitals had spent more than US$ 3 on NCCCP implementation. We were able to demonstrate that investments in cancer care and research appeared to be associated with at least one of three strategic goals that hospital executive management valued most from their affiliation with NCI: increased cancer patient volume. We were unable to demonstrate an association between investments and changes in market share or recruitment of key cancer physicians.

Increased cancer patient volume seemed to be the outcome with the clearest association to total NCCCP investments. This measurable change could be because it is the outcome most likely to change in the shortest period of time. Since the time period examined was only three years, it is unlikely that sufficient time had passed to expect a change to be demonstrated in data specific to Medicare market share. Increases in key cancer physicians affiliated with the participating hospitals were reported for nine of the 10 lead sites. Because all but two hospitals participating in the NCCCP pilot rely primarily on private practice physicians, this increase represents substantial growth, particularly for specific hospitals (e.g. increases of as many as 19 or 35 physicians). Without knowing the extent to which changes in physician numbers had occurred prior to NCCCP, it is impossible to link the program’s efforts to this particular outcome. However, we know anecdotally that two suburban hospitals recruited new cancer centre directors to their areas who were also physicians with national reputations as a direct result of being a NCCCP site, and two rural hospitals and six suburban hospitals were able to recruit subspecialists (e.g. breast surgeon, gynaecological oncologist) to their area for the first time. These respondents, who were new to the hospital, shared in interviews that the hospital’s partnership with NCI and their clear commitment to the cancer service line were the primary reasons they had opted to accept a position there. In addition, most hospitals reported having more physicians engaged in ongoing care coordination because of the extra efforts being made through NCCCP implementation (e.g. multidisciplinary care conferences).

Although the NCCCP evaluation was a comprehensive, mixed-method design, there are limitations to the data used for the analyses that should be considered. One obvious challenge is the limited number of sites, although extensive data were collected and several sources summarized for the 10 sites presented here. While findings from the evaluation of the NCCCP are limited to a sample size of these 10, our findings provide a unique opportunity to assess whether public-private partnerships, as vehicles to leverage local investment, can mutually benefit NCI, the participating institutions, and the communities they serve. Timing of data collection was also a factor since presented data were collected at slightly different points in time, making explanations of findings challenging (e.g. cost data were collected at a different time of year than interviews were conducted). Another element of time that is important to consider is that each hospital’s cancer program was in a different phase of their development, with some being clear leaders in their local markets at the start of NCCCP while others were not. Similarly, some hospitals had fully integrated cancer service lines with a medical director and clinical trials program while others did not have that capacity at the start. Therefore, we assessed the percent change in each outcome for each individual hospital (i.e. comparing their post-measure to their own pre-measure, not comparing them to other hospitals) and rank ordered them within two groups, producing a relatively crude estimate of change over time. With a larger sample size, we would have been able to perform more sophisticated analyses to explore patterns in investments and the three outcomes. Finally, because all hospitals in the study were participating in the NCCCP, and many were making other cancer care changes contemporaneous to the program, full attribution of outcomes to the NCCCP is not possible. Still, these findings provide a first look at the motivations of hospital executives to commit to programs such as the NCCCP, and the types of changes that might be expected in a relatively short period of time.

## Conclusions

The NCCCP was a pilot program implemented on a relatively small scale (in 16 hospitals) to test the feasibility of the concept of the NCCCP as a public-private partnership to more quickly diffuse the rapidly changing science around cancer care into community settings. The overall evaluation was intended to provide a formative evaluation of what seemed to work well and what should be improved upon to enhance the NCCCP model. In this way, the evaluation findings, along with the program, had to evolve over time because so many lessons were learned during each phase of implementation.

The scientific landscape (e.g. advances in genomics), healthcare reform, and policy and socioeconomic issues all influenced the rationale for creating the NCCCP as a public-private partnership [1]. NCI leveraged its funding to fulfil important research goals through partnerships with community hospitals. These partnerships benefited the hospitals through their affiliations with NCI, and the access to NCI staff and resources that these affiliations afforded. In exchange, the NCI realized the benefits of hospitals’ co-investments and efforts to support mutually beneficial goals. The partnerships required collaboration with the management of the participating hospitals, since management controls the resources of the institution and must make decisions to navigate competitive and policy-driven environments. Clinician participation in the NCCCP was clearly important, but gaining and sustaining hospitals’ commitment relied critically on the existence of a strategic case for involvement in the NCCCP. This took the form of expected patient volume and market share increases, as well as enhanced opportunities for physician recruitment.

Our cost study shows that private investment in cancer service lines translated into an average of US$ 3 in hospital funding for every US$ 1 provided by NCI. This level of investment alone demonstrates how much of a catalyst the NCCCP was in driving the hospitals to focus on advancing science in their local areas. We have reported elsewhere that the evaluation results indicate that most of the participating hospitals were able to expand their cancer programs and research infrastructures [13-16], increase evidence-based care [17], increase physician engagement [18,19], and invest significant matching funds [20]. However, with regards to executives’ reported strategic goals, this study showed that levels of financial investment were only associated with increases in cancer patient volume over the 3-year period. Increasing market share may take more time as it is highly dependent on the competitive environment. Recruitment of key cancer physicians was seen across all but one of the participating hospitals, regardless of levels of matching investment. Results of qualitative interviews suggest that physician recruitment may be driven more by the NCI affiliation and the perceived commitment of the hospital to cancer care, than by actual levels of spending, at least in the short-term.

Importantly, implementation of the NCCCP occurred within the context of the worst economic downturn since the Great Depression, which added to the stress of hospitals investing in this public-private partnership. Moreover, it coincided with the early years of the Affordable Care Act, when significant market shifts were occurring and reimbursement methods were changing. The multiple dimensions of the turbulent environment faced by NCI, as well as the management and clinicians in the participating community hospitals were a challenge. Yet, despite these challenges, the participating hospitals found value sufficient to justify their investment, thus illustrating the potential of public-private partnerships with aligned goals to leverage federal funds to achieve important policy objectives.

In the years ahead, initiatives to advance value-based care will increase given the major changes underway as a result of the Affordable Care Act’s passage in 2009. Based on the experience of the NCCCP, there is no doubt that there is much that government can accomplish in collaboration with healthcare organizations to manage disruptive change and to advance science and improve healthcare in the community. Despite its small sample size, the NCCCP evaluation provides an opportunity to document the lessons learned from the program and ensure that future programs are informed by these efforts. This study also contributes to existing research on the business case for quality by expanding the concept to the strategic case. Our study is a first step in determining how to assess the ‘strategic case’ for such a partnership, and helps to inform the types of outcomes that can realistically be expected to occur in a relatively short time-frame. Still, more needs to be understood about how best to facilitate these public-private partnerships and under what circumstances they lead to outcomes desired by all partners involved.

## Availability of supporting data

The data set(s) supporting results of this article and collected from the participating hospitals are not available due to constraints of our Data Use Agreements. Medicare market share data are readily available via www.cms.gov  (Accessed 10-1-15).

## Endnotes

^a^For more details on the methods and data sources used in the overall evaluation, see [12].

^b^For more details on the micro-cost study methodology, see [20].

^c^Note that the baseline patient volume count for calendar year 2006 was provided by sites in their initial proposals for funding.

^d^Available from the Centers for Medicare & Medicaid Services

## Abbreviations

CY: Calendar year
CEO: Chief Executive Officer
COO: Chief Operating Officer
CFO: Chief Financial Officer
FY: Fiscal year
NCCCP: National Cancer Institute’s Community Cancer Centers Program
NCI: National Cancer Institute
RTI: RTI International
